# Acupuncture in cancer care: a narrative review

**DOI:** 10.1590/1806-9282.2024S101

**Published:** 2024-06-07

**Authors:** André Wan Wen Tsai, Eduardo D'Alessandro, Sidney Brandão, João Bosco Guerreiro, Ricardo Morad Bassetto, Janete Shatkoski Bandeira, Marcus Yu Bin Pai, Adriano Höhl, Alexandre Valotta da Silva, Fernando Mendes Sant'Anna

**Affiliations:** 1 Brazilian Medical College of Acupuncture – São Paulo (SP), Brazil.; 2 Universidade de São Paulo, Clinical Hospital, Faculty of Medicine – São Paulo (SP), Brazil.; 3 The São Leopoldo Mandic Faculty of Medicine – São Paulo (SP), Brazil.; 4 Faculty of Medicine of São José do Rio Preto – São José do Rio Preto (SP), Brazil.; 5 Institute of Scientific Chinese Medicine – São Paulo (SP), Brazil.; 6 Neurofunctional Acupuncture Study Group – Porto Alegre (RS), Brazil.; 7 Santa Casa de Bragança Paulista – São Paulo (SP), Brazil.; 8 Universidade Federal do Rio de Janeiro – Macaé (RJ), Brazil.

## INTRODUCTION

Cancer is the main public health problem worldwide, being one of the main causes of death and, consequently, one of the main barriers to the increase in life expectancy^1^. The impact of cancer, based on estimates by the Global Cancer Observatory (Globocan)^2^, prepared by the International Agency for Research on Cancer (IARC), points out that there were 19.3 million new cases of cancer in 2020 worldwide. One in five individuals will have cancer during their lifetime^2,3^. Notably, 10 main types of cancer represent more than 60% of all new cases. Female breast cancer is the most common cancer worldwide, with 2.3 million (11.7%) new cases, followed by lung cancer, with 2.2 million (11.4%); colon and rectum, with 1.9 million (10.0%); prostate, with 1.4 million (7.3%); and non-melanoma skin, with 1.2 million (6.2%) new cases. In Brazil, the estimate for the 3-year period from 2023 to 2025 indicates that there will be 704,000 new cases of cancer, 483,000 if cases of non-melanoma skin cancer are excluded^1^.

Systemic treatment or combined therapies are commonly used for cancer patients, among which palliative care may be used for the alleviation of cancer-related symptoms, side effects related to conventional treatment, as well as the healthcare of cancer survivors^4,5^.

Acupuncture, a therapy originating from the system of traditional Chinese medicine (TCM), has been in use for at least 2500 years. Fine needles are inserted and stimulated, either manually or electrically, to treat specific symptoms or health conditions. According to the World Health Organization (WHO), acupuncture is used in at least 103 countries, some of which have established regulations for providers^6,7^. In the United States, about 3.5 million adults receive acupuncture for each ear^4,7^.

Acupuncture is also widely used for palliative and supportive care for cancer patients, and it has not only been limited to manual acupuncture but has also incorporated electrical acupuncture, auricular acupuncture, laser acupuncture, etc^8–10^. Based on a large number of recommendations made by clinical practice guideline development groups and expert groups from 13 countries, acupuncture has been recommended for chemotherapy-induced and post-operative nausea and vomiting, cancer pain, fatigue, insomnia, xerostomia, hot flashes, lymphoedema, and chemotherapy-induced neuropathy, as well as to improve the quality of life (QoL) of these patients^11,12^. In addition, acupuncture has been considered a safe therapy for cancer patients when practiced by qualified practitioners, and although mild adverse events (AE) like dizziness, fatigue, and nausea may appear, they generally improve without any extra measures^13,14^.

In this narrative literature review, we will briefly address the main clinical evidence for the use of acupuncture in cancer patients and cancer survivors, ending with aspects related to the safety of the procedure and implications for healthcare policy in these patients.

## CLINICAL EVIDENCE FOR ACUPUNCTURE IN CANCER CARE

Emerging research has found promising evidence for the role of acupuncture in oncology, especially to control symptoms where existing standard options remain a challenge. Three relatively recent reviews^6,11,12^ synthesized data across several systematic reviews (SR) for effects on symptoms such as pain, fatigue, hot flashes, nausea/vomiting, xerostomia, insomnia, lymphoedema, bone marrow suppression, and QoL (central illustration).

In the following sections, we will show a summary of the data presented in these and other reviews on the above-mentioned cancer-related conditions.

### Pain

The prevalence of pain in cancer patients varies widely depending on the type of cancer and the stage of the disease. A SR and meta-analysis published in 2016 analyzed 117 articles that addressed the prevalence of pain in cancer patients, which was approximately 39.3% [95% confidence interval (CI) 33.3–45.3%] after the end of curative treatment, 55% (95%CI 45.9–64.2%) during treatment, and 66.4% (95%CI 58.1–74.7%) in cases of advanced disease, with a combined prevalence in all stages of treatment of 50.7% (95%CI 37.2–64.1%)^15^. The same study also analyzed 52 articles that reported pain intensity and indicated that moderate to severe pain is present in 38% (95%CI 32.8–43.3%) of patients. In another review, around 90% of cancer patients in advanced stages have moderate to severe pain, and in those with early and intermediate stages, the prevalence reaches 40%^16^. Unfortunately, management ends up being inadequate for up to 70% of individuals with cancer, damaging their QoL^16^.

Pain in this context has different origins, including factors related to the presence of the tumor itself and the treatments performed. Nociceptive pain is often caused by the compression or invasion of tissues by tumor cells. Neuropathic pain can occur due to injuries induced by surgical, radiotherapy, and/or chemotherapy treatments. Clinical studies highlight the heterogeneous nature of cancer pain, with mixed pain being considered the most common^17^.

The use of acupuncture in the treatment of pain in oncology has been investigated over the last few decades, making it possible to carry out some reviews. However, the methodological quality of the publications still hinders the analysis of their value. A review published in 2017 included 29 articles that addressed patients over 18 years and indicated that acupuncture has a moderate effect on pain induced by the tumor lesion as well as by surgical treatments (-0.7 and −0.4; 95%CI −0.94 to −0.48 and −0.69 to −0.1, respectively)^18^.

Despite the doubts that still linger on the subject, based on the evidence available in the literature, the study group "*International Trustworthy Traditional Chinese Medicine Recommendations (TCM Recs) Working Group*" published an article with three recommendations in 2022^19^:

1. Strong recommendation for the use of acupuncture when not treating moderate to severe pain in cancer patients.
2. Weak recommendation for the combination of acupuncture and acupressure in pain management treatments with the aim of reducing the use of opioids and the incidence of side effects induced by such drugs in patients with moderate to severe pain who are using analgesics.
3. Strong recommendation for the use of acupuncture to relieve arthralgia induced by aromatase inhibitors in patients with breast cancer.

A SR conducted by Yang et al., concluded that acupuncture is a safe and effective treatment to reduce the intensity of pain in cancer patients under palliative care^20^.

Therefore, we understand that pain in oncology is a significant concern that requires a multidisciplinary approach to its management. Acupuncture has been shown to be an effective option, but its usefulness must be evaluated considering the context of the disease, its staging, and the etiology of the pain.

### Fatigue

Cancer-related fatigue (CRF) is one of the most frequent symptoms of cancer patients and cancer survivors. Although related to the illness process, the main reason for it is the treatment, like surgery, radiation therapy, and the most common chemotherapy^21^.

In recent years, much research has been carried out to validate the efficacy of acupuncture for this disorder. From 2013 to 2023, 21 SR and 12 meta-analyses (MA) were also performed. However, 15 SR and 8 MA were performed only for acupuncture, and in 6 SR and 4 MA researchers observed other kinds of complementary and integrative therapeutics, including acupuncture.

He et al.^22^ found 7 studies involving 804 patients and concluded that despite the few high-quality randomized controlled trials (RCTs), acupuncture appears to be an efficacious method for CRF. In other SR, Posadzki et al.^23^ observed four trials in seven that showed effectiveness against *sham* or usual care, although most of them were small pilot studies, and concluded that it remained unclear whether the results were due to specific effects of acupuncture. Finnegan-John et al.^24^, analyzing 20 studies using complementary therapies for the management of CRF, concluded that acupuncture may reduce this symptom following cancer treatments.

Choi et al.^25^, analyzing 12 studies with 1,084 participants with breast cancer, observed that acupuncture was more effective than *sham*, usual care, or waiting list. They called attention to the fact that most of the studies were of low quality, mainly because of the small samples. Also in 2022, the same author^25^, in an overview of SR and MA with 10 SRs, 160 RCTs, and 14,392 patients, declared that most of the SRs reached the potential benefits of acupuncture for CRF, despite the methodological quality of most of them was low. In the same year, Zhang et al.^12^ published an overview of SR for many cancer-related conditions, comprising 7 SRs and 18 RCTs, which showed acupuncture's efficacy in controlling CRF. Tian et al.^26^, in a Bayesian network MA and SR where 34 RCTs and 2,632 participants were included, comparing acupuncture with *sham* interventions, usual care, or waiting lists, showed that acupuncture was effective and safe for CRF treatment.

### Hot flashes

Hot flashes are a subjective symptom associated with objective signs of cutaneous vasodilation and a subsequent drop in core temperature with sweating, flushing, palpitations, anxiety, panic, and irritability, which appear in women with hypoestrogenism during climacteric and menopause periods. Among survivors of gynecological cancer, there is an increase in the prevalence of symptoms by up to 35%, worsening the QoL of these patients^27^.

Many factors have been linked to loss of control of temperature regulation in the hypothalamus, including pituitary hormones, hormone-releasing factors, gonadotropins, and neurohumoral pathways. Menopause estrogen reductions are associated with decreased endorphin and 5-hydroxytryptamine (5-HT) levels and increased 5-HT receptors. This results in a loss of the feedback mechanism of increased norepinephrine production, which can reduce the thermoneutral zone and thus increase the likelihood of flushing. Therefore, any substance that increases 5-HT, estrogen, endorphins, or decreases norepinephrine can widen the thermoneutral zone and, therefore, reduce hot flashes^28^.

Non-hormonal treatments, with selective serotonin reuptake inhibitors, serotonin reuptake inhibitors, norepinephrine, and gabapentin, provide partial relief to women, but with very frequent side effects, causing treatment abandonment^6,29^.

Acupuncture has been recommended in the treatment of cancer patients due to its safety, high accessibility, and minimal risk of causing endometrial problems. Its action in increasing endorphin activity and neurotransmitter levels (serotonin, dopamine, and noradrenaline, in addition to metenkephalin and substance P) has already been proven, which may be associated with the modulation of thermoregulation in the hypothalamus and the neutralization of vasomotor symptoms^30^.

Despite difficulties in scientific methodology in acupuncture trials, evidence has grown in favor of its use as an adjuvant treatment for hot flashes. Lund et al.^31^ report that standardized, brief acupuncture treatment can produce a rapid and clinically relevant reduction in moderate to severe menopausal symptoms during a six-week intervention. Li et al.^32^ state that the effectiveness of the combination acupuncture and electroacupuncture was superior to that of *sham* acupuncture and significantly superior to placebo pills, with electroacupuncture being superior to traditional acupuncture, significantly superior to *sham* acupuncture, and comparable to the results of selective serotonin inhibitors, serotonin reuptake/selective serotonin-norepinephrine reuptake inhibitors, and neuroleptic agents, with reports in the literature of therapeutic effects that persisted for six months or more and did not require continued treatment^29,33^.

### Nausea/vomiting

Nausea and vomiting are very common conditions in the universe of gastrointestinal dysfunctions or pathologies. In general, they are triggered by emetic stimuli not only through the central nervous system (CNS) and/or peripheral nervous system (PNS), affected by toxins, drugs, bacteria, viruses, or fungi, enterally or parenterally, but also through the skin and respiratory system^34,35^.

Noxious stimuli, or those recognized as such, initiate the emetic reflex coordinated by the dorsal vagal complex in the brain stem, composed of the area postrema, the nucleus of the solitary tract, and the dorsal motor nucleus of the vagus^34,35^, frequent therapeutic targets of acupuncture, whether systemic or auricular.

Stimulation of deep tissues through afferent sensory nerves is the initial event that leads to the activation of pathways involved in sensory modulation in the CNS and autonomic regulation^6^.

The therapeutic potential that emerges from some known mechanisms, pathways of action, and therapeutic targets of acupuncture is effective in a relatively robust body of clinical research. Data related to post-surgical nausea and vomiting suggest a biological effect through stimulation of the acupuncture point with a needle and electrical stimulation and demonstrate benefits in controlling vomiting induced by chemotherapy^36^.

In a SR and MA published in 2022, Xi et al., considered acupuncture to be promising, both post-surgery and post-chemotherapy, to treat symptoms related to cancer. Acupuncture, in this review, in relation to side effects caused by chemotherapy or radiotherapy, such as nausea and vomiting specifically, proved to be a viable, safe, and cost-effective alternative^37^.

Considering that the aim of cancer treatment is not only to eliminate the tumor lesion but also to promote the patient's QoL during treatment and beyond, therapeutic approaches that contribute to this aspect are obviously welcome. Significantly, in this sense, acupuncture has proven particularly useful in controlling gastrointestinal dysfunctions^38^.

We can conclude that acupuncture can be an option for cancer survivors with relevant suffering conditions, including nausea and vomiting, that acutely affect the QoL and nutritional capacity of these individuals^12^.

### Insomnia

About a third to even more than half of cancer patients have insomnia^39–42^. The prevalence of insomnia differs across different types of cancer but is higher than in the general population^43^. A review by Choi et al.^44^ including six RCTs, demonstrated that acupuncture was superior to *sham* acupuncture, drugs, or hormone therapy for insomnia treatment. A recent RCT with blinded data collectors and using the Standards for Reporting Interventions in Clinical Trials of Acupuncture (STRICTA) guidelines found a significant benefit of acupuncture in improving sleep quality in women with climacteric-like symptoms associated with breast cancer treatment^45^.

### Xerostomia

For patients with head and neck cancer undergoing combined chemoradiation treatment, xerostomia is one of the most common and debilitating side effects^6^. Recent reviews have analyzed the effect of acupuncture in the treatment of xerostomia in cancer patients^46,47^. Although several studies present a risk of bias, acupuncture had a favorable effect on patient-reported xerostomia compared with *sham* acupuncture or no treatment. The most relevant common finding in more recent papers was that acupuncture treatment was significantly associated with less severe patient-reported symptoms of salivary impairment compared with standard supportive measures up to 1 year from the end of treatment^47^.

### Lymphoedema

Acupuncture has been explored as a potential treatment for breast cancer-related lymphoedema (BCRL). BCRL is a common complication after breast cancer treatment, involving the accumulation of lymph fluid and swelling in the arm. The mechanisms by which acupuncture may help treat BCRL are not fully understood but likely involve enhancement of lymphatic circulation. According to TCM principles, acupuncture can restore the flow of *qi* and blood, thereby reducing stagnation and swelling. From a Western perspective, acupuncture may reduce inflammation, induce the release of neuropeptides, and modulate autonomic nervous system activity to improve lymphatic drainage^48^.

Evidence for the efficacy of acupuncture in BCRL has been examined in several RCTs. A SR by Chien et al.^48^ included 6 trials with 178 patients and found acupuncture did not significantly reduce arm circumference versus control but appeared safe. The heterogeneity of acupuncture techniques, outcome measures, and lack of symptom monitoring were limitations. A larger trial by Bao et al.^49^ with 73 patients did not find a significant difference in arm circumference or bioimpedance between acupuncture and waitlist control. However, a 2016 study by Yao et al.^50^ did report significantly greater improvement in arm circumference and range of motion with acupuncture versus medication. Overall, existing studies show promising results for acupuncture but are limited by small sample sizes.

While acupuncture may not significantly decrease limb size, it may still provide subjective symptom relief in BCRL. A pilot study by Cassileth et al.^51^ found acupuncture was associated with symptom improvement in 9 patients. A trial by Jeong et al.^52^ showed reduced pain and improved QoL with acupuncture in 9 patients. Though limited by the lack of control groups, these studies indicate acupuncture could benefit patient-reported symptoms. Larger studies using validated symptom surveys are needed.

Accumulating evidence shows that acupuncture is safe for BCRL patients, but uncertainty remains regarding its efficacy in limb size reduction. Potential benefits for patient symptoms have been reported, though high-quality randomized trials are still needed. Future research should utilize larger sample sizes, standardized acupuncture protocols, objective swelling assessments, and patient-reported outcomes. Elucidating the mechanisms of acupuncture in BCRL may help optimize techniques. Overall, acupuncture shows promise for integration into multimodal BCRL treatment.

### Bone marrow suppression

Acupuncture has emerged as a promising supportive therapy to help mitigate myelosuppression from chemotherapy. Animal studies show acupuncture helps to regulate cytokines, growth factors, and proteins vital to hematopoiesis and immune function in models of chemotherapy-induced leukopenia and cytopenia^53,54^. Clinical studies, including a few randomized trials, indicate acupuncture reduces the severity of leukopenia and related side effects like fatigue in cancer patients undergoing chemotherapy^55,56^.

Proposed mechanisms include immunomodulation, anti-inflammatory effects, and regulating hematopoiesis. Specific actions include enhancing DNA repair in bone marrow cells^54^, increasing hematopoietic cytokines like granulocyte-macrophage colony-stimulating factor (GM-CSF)^53^, stimulating hematopoietic stem cell proliferation^54^, and modulating regulatory proteins like cyclin D1 that influence marrow cell cycling^54^. Acupuncture may also improve the bone marrow microenvironment by promoting angiogenesis and energy metabolism.

Clinical research indicates acupuncture is well-tolerated and improves symptoms like fatigue and QoL in chemotherapy patients^56^. Adding acupuncture to usual care significantly reduced the severity of leukopenia^55^. While animal models suggest immune and hematopoietic effects, rigorous evidence in humans is needed. There is also debate around optimal acupuncture points and techniques for managing cytopenia.

### Quality of life

Acupuncture has been shown to be effective in treating physical and mental conditions related to cancer patients' health. The QoL of these patients is often compromised, not only by the symptoms of the disease itself but also by the side effects of therapeutic interventions, whether radiotherapy or chemotherapy.

There are a variety of scales for judging the improvement of QoL. Two recent reviews focused on QoL^57,58^. The first one included only two RCTs of low quality and concluded that acupuncture was superior to conventional treatment^57^. The second, comprising 14 RCTs and 1,225 participants, pooled the results of different scales to evaluate the improvement of QoL and showed that acupuncture improved overall QoL compared with *sham* or no intervention.

## SAFETY OF ACUPUNCTURE IN CANCER CARE

Needling performed in an acupuncture session is generally considered safe, with publications indicating a higher incidence restricted to low-risk AE and very rare serious events when performed by trained practitioners^59,60^. However, some particularities of the oncological context must be observed.

The greatest concern is the greater risk of infections and bleeding, since the interventions used to treat this population often alter both their immunity and clotting factors and modify the usual anatomy with tissue resections and prosthetic implants. Another point of attention is adequate communication between the acupuncturist, the team that conducts the oncological treatment, the patient, and their loved ones to avoid conflicts and guarantee the integration of this therapy as a complement and not a replacement for the usual treatment.

A recent SR and MA managed to include 65 articles that addressed the occurrence of AE in this context^14^. Their results indicate that there is no increase in the incidence of AE when comparing groups undergoing acupuncture and their controls (*sham* or active) but indicate a greater risk of the occurrence of low-risk events such as small bleeding, hematomas, pain at the needling site, and syncope when compared with groups undergoing usual treatment. The authors' conclusion, however, highlights the great heterogeneity in the quality of the data analyzed and suggests greater attention to the method used when reporting the AE that occurred.

Based on the data available in the literature and the experience of these authors, we suggest avoiding needling in situations of neutropenia with neutrophils below 1,000/mm^3^, thrombocytopenia with platelets below 25,000/mm^3^, and changes in clotting times with an international normalized ratio (INR)>2.0 or partial thromboplastin time (PTT)>60 s. Regarding the puncture site, we suggest avoiding needling adjacent to places with surgical synthesis material, metal rods, plates, and similar, the presence of tumors, and, in the case of bone lesions in the spine, avoiding needling in the muscular layer adjacent to the puncture site due to the risk of altering any muscle contractions that may be keeping the spine stable.

## IMPLICATIONS FOR CLINICAL PRACTICE

For clinical practice, some SR support the use of acupuncture for cancer-related pain, CRF, breast cancer-related hot flashes, nausea, and vomiting, which is in line with clinical practice guidelines^11,19^.

Clinical studies should be recommended on conditions that commonly use acupuncture in routine clinics, but lack high-quality or well-reported evidence, such as xerostomia, lymphoedema, and insomnia. Clinical trials are strongly recommended to be reported by the CONSORT Statement and its extension to acupuncture trials (STRICTA)^61^, to maintain a high methodological quality.

For SR and meta-analysis, many have been conducted in the last few years for different cancer-related conditions. It is recommended that the authors who wish to perform a SR or a MA stick to the Preferred Reported Items for Systematic Reviews and Meta-Analyses (PRISMA)^62^, registering protocol before conducting, providing an exclusion list if possible, and reporting by acknowledged criteria.

## CONCLUSION

This narrative review showed that acupuncture can be used for various cancer-related conditions, such as cancer-related pain, CRF, insomnia, QoL, nausea and vomiting, bone marrow suppression, lymphoedema, and xerostomia. It is a safe method, with no serious adverse effects reported. Future reviews reporting according to the acknowledged reporting standards are recommended to improve the quality of evidence.
